# Supplementary material for: Microfluidic Diffusion Sizing Applied to the Study of Natural Products and Extracts That Modulate the SARS-CoV-2 Spike RBD/ACE2 Interaction
Source: Molecules. 2023 Dec 13;28(24):8072. doi: 10.3390/molecules28248072 (PMC10745392; doi:10.3390/molecules28248072)
Supplement: Supplementary file 1 [file molecules-28-08072-s001.zip › molecules-2719130-supplementary.pdf]

Supplementary Materials
"Microfluidic Diffusional Sizing applied to the study of natural products and extracts that modulate the SARS-CoV-2 SpikeRBD/ACE2 interaction"

Method validation

1. *Selectivity*

To evaluate the selectivity of the developed MDS method, 3 samples were analyzed: $SpikeRBD_{labelled}$ at 507 nM, ACE2 at 750 nM, and a mix PBS:Tween 20 (99.95:0.05, v/v) (=PBS-T) as Blank. Each analysis was performed in 3 replicates. We then compared the electropherograms from each analysis.

Figure S1 Electropherograms of the 3 samples: Fluorescence-labelled SpikeRBD ($SpikeRBD_{labelled}$), concentration = 507 nM; ACE2, concentration = 750 nM ; PBS-T.

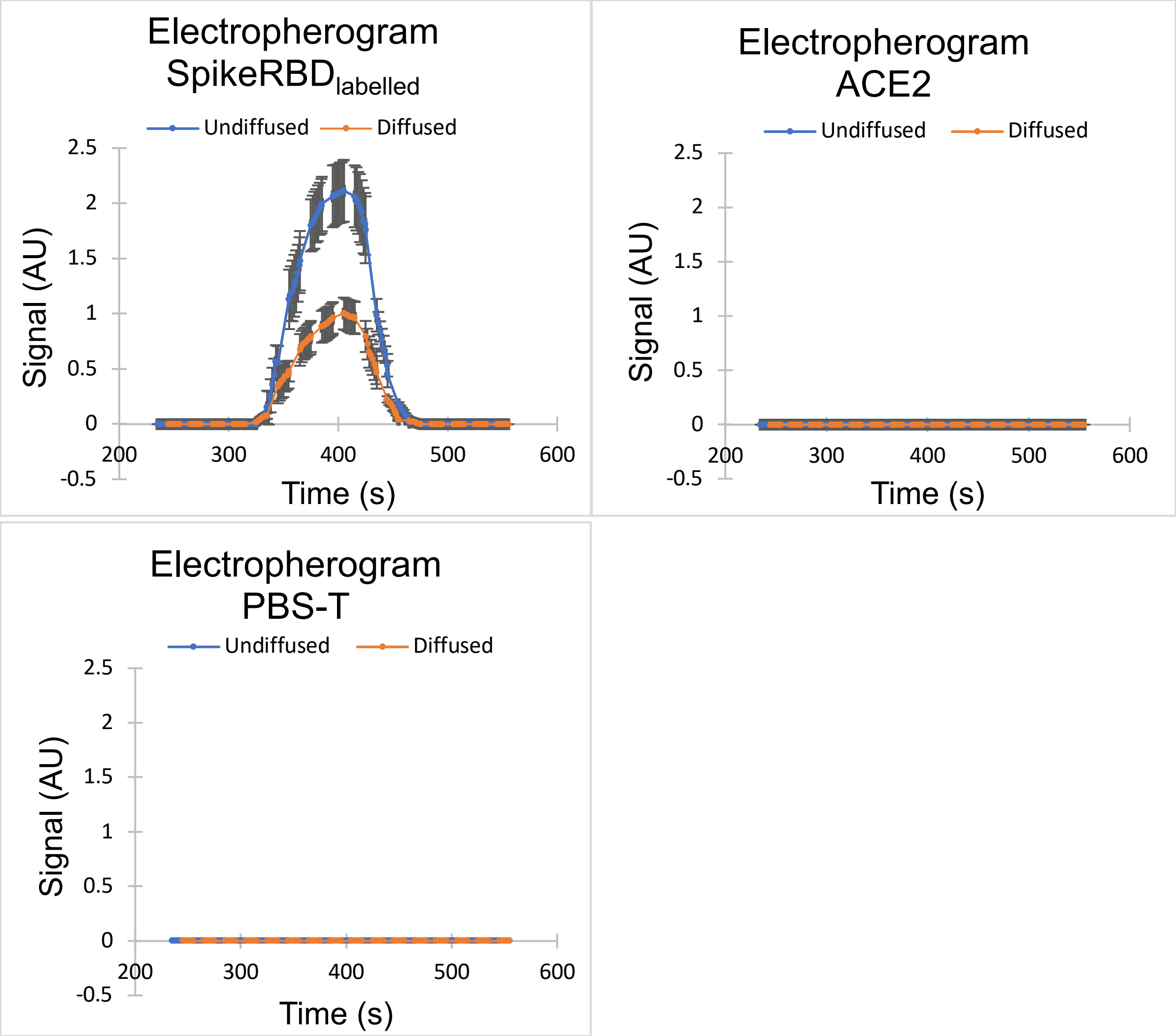

Microfluidic Diffusional Sizing: evolution of fluorescence in the diffused and undiffused channels as a function of time (mean ± SD; n = 3). Arbitrary unit abbreviated as AU.

These 3 electropherograms (Figure S1) indicate that, of these 3 samples, only the $SpikeRBD_{labelled}$ yields a detectable signal, allowing a determination of R_h. The method is then selective towards the fluorescent analyte. Also, at the working wavelengths of the apparatus ($\lambda_{Excitation}$, 630 nm; $\lambda_{Emission}$, 694 nm), there is a very low number of natural products that will fluoresce or quench the incident and emitted radiation («*Quenching*»). For all subsequent analyses, this was systematically checked by montoring the signal.

2. *Reproducibility*

Since the technology is relatively recent, the reproducibility of disposable chips was assessed, as well as their limits. To do this, the following 3 tests were carried out:

- Use of a single chip to measure each data point
  (Analysis of the fluorescently labelled SpikeRBD = $SpikeRBD_{labelled}$)

Table S1 One chip per data point – Reproducibility

Sample	t° of chip (°C)	[$SpikeRBD_{labelled}$] (nM)	Hydrodynamic radius (R_h) (nm)
Labelling 1 – 2022/01/19 (n = 3)	23.1	50	3.03
	22.3	50	2.94
	21.1	50	2.74
Labelling 2 – 2022/02/18 (n = 3)	22.6	50	2.76
	23.3	50	2.93
	21.9	50	2.82
Mean			2.87
Standard Deviation			0.11
Relative Standard Deviation (%)			3.99

Measure of SpikeRBD R_h using 1 chip per data point. Mean, standard deviation, and relative standard deviation.

- Use of a single chip to repeatedly measure data points
  (Analysis of the fluorescently labelled SpikeRBD = $SpikeRBD_{labelled}$)

Between each measurement, the remaining traces of the previous sample were carefully removed using a micropipette.

Table S2 One chip to measure several data points

Sample	[$SpikeRBD_{labelled}$] (nM)	R_h (nm)
Chip 1 – measure (n = 8)	507	3.07
	507	3.05
	507	1.98
	507	3.14
	507	2.80
	507	1.36
	507	2.93
	507	4.41
Mean		2.84
Standard deviation		0.89
Relative Standard Deviation (%)		31.4

Measure of SpikeRBD R_h using 1 chip for all data points. Mean, standard deviation, and relative standard deviation.

A very low reproducibility was observed for these repeated measurements, although the analyses were performed at a 10 times higher concentration, compared to Table S1. This is probably explained by the presence of air bubbles in the microfluidic channels.

- <u>Use of a single chip to analyze a full affinity curve to determine K_D</u>

All the samples required for the K_D determination of the SpikeRBD/ACE2 protein complex were injected on a single chip. Each data point was analyzed in 3 replicates and measurements were performed in ascending order of ACE2 concentrations. Between each measurement, the remaining traces of the previous sample were carefully removed using a micropipette.

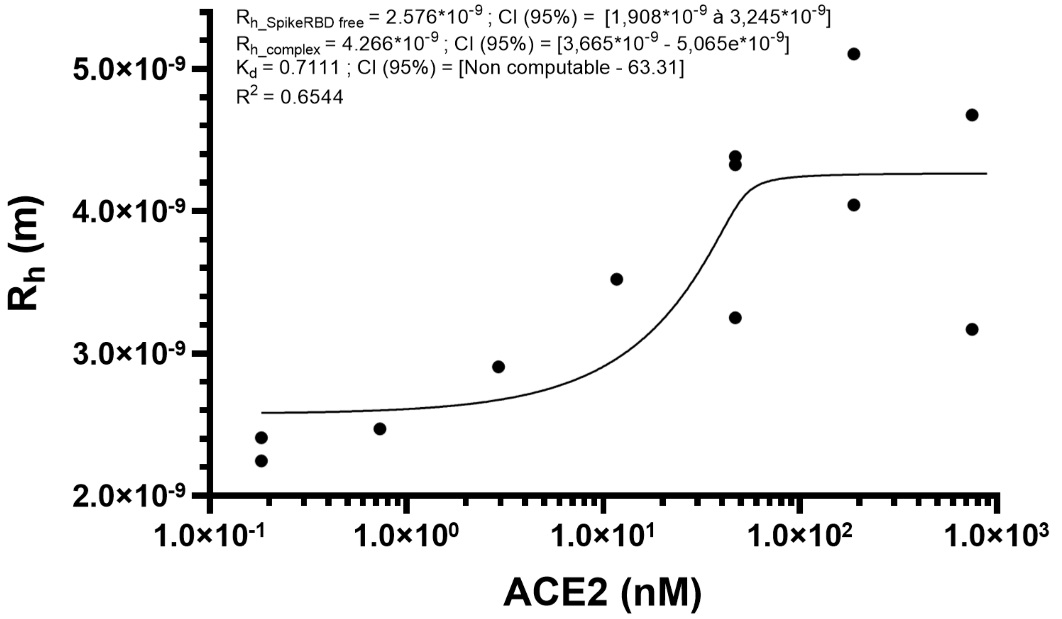

Figure S2 : Microfluidic Difusional Sizing determination of K_D for the SpikeRBD (20 nM)/ ACE2 complex (DMSO, 1% v/v; room t °). R_h as a function of ACE2 concentration. [ACE2], 180 pM-750 nM; [SpikeRBD]; 20 nM; mean ± standard deviation (n = 3). All data points were obtained on a single chip: ACE2 concentrations were injected in the ascending order, remaining traces of the previous sample being carefully removed using a micropipette.

It should be noted that 3 replicates measurements were feasible for only one point: [ACE2] = 46.9 nM; for the other replicates, an error message was displayed, indicating an instrumental error (? Microbubbles probably).

It is not reliable to use only one chip to determine the K_D, which is consistent with the conclusion of the previous test.

All subsequent experiments were performed using 1 chip/R_h measurement.

3. *Accuracy (accuracy) in the measurement of the hydrodynamic radius of SpikeRBD$_{labelled}$*

A theoretical value of SpikeRBD$_{labelled}$ R_h was computed with a software proposed by Fluidic Analytics (https://www.fluidic.com/calculators-page/), by encoding the molecular weight of the protein (31.25 kDa), and selecting a *"Folded (globular)"* state. The theoretical R_h was estimated at 2.72 nm, based on the Stokes-Einstein equation, and on proprietary calibration curves [1, 2].

To determine the accuracy of MDS measurements, these were compared to the theoretical R_h (Table S3).

Table S3 Determination of R_h in different conditions – Comparison to theoretical R_h

Sample	t° of chip (°C)	[SpikeRBD$_{labelled}$] (nM)	R_h (nm)	Relative R_h (%)
Labelling 1 – PBS-T (n = 3)	23.1	50	3.03	111.40
	22.3	50	2.94	108.09
	21.1	50	2.74	100.74
Labelling 2 – PBS-T (n = 3)	22.6	50	2.76	101.47
	23.3	50	2.93	107.72
	21.9	50	2.82	103.68
Protein dissolved in MiliQ water	22.8	20	3.01	110.66
Protein dissolved in PBS-T	23.4	20	2.92	107.35
	23.8	5	2.90	106.69
	23.8	5	2.76	101.62
	26.1	5	2.49	91.43
Protein dissolved in DMSO 1 %, v/v	25.2	20	3.06	112.61
	25.6	20	2.89	106.18
Mean			2.87	105.4
Standard Deviation			0.15	5.7

The mean R_h was measured at 2.87 ± 0.15 nm, which represents a recovery rate of the theoretical value of 105.4 ± 5.7 %.

4. *Precision*

- Inter-day precision on the hydrodynamic radius of $SpikeRBD_{labelled}$

Table S4 Inter-day variability of $SpikeRBD_{labelled}$ R_h -

Context of the measure	Date	[$SpikeRBD_{labelled}$] (nM)	Mean R_h (nm)
Labelling 1	2022/01/19	50	2.900
Labelling 2	2022/02/18	50	2.840
Protein dissolved in PBS-T	2022/01/20	5	2.902
	2022/01/21	5	2.764
	2022/01/24	5	2.487
Protein dissolved in DMSO 1 % v/v	2022/02/21	20	3.063
	2022/02/21	20	2.888
Mean			2.83
Standard Deviation			0.18
Relative Standard Deviation (%)			6.27

.

The inter-day (2.83 ± 0.18 nm) and within-day precision (2.87 ± 0.11; Table S1) are of the same order.

- Intra-day precision on the hydrodynamic radius of the complex $SpikeRBD_{labelled}$/ACE2

Table S5 Intra-day and total variability of the R_h for the complex $SpikeRBD_{labelled}$/ACE2

[$SpikeRBD_{labelled}$] (nM)	20	20	20
[ACE2] (nM)	0.183	20	750
R_h1 (nM) - Curve 1	3.105	3.580	4.374
R_h2 (nM) - Curve 1	3.019	3.309	4.210
R_h4 (nM) - Curve 2	2.679	3.294	4.391
R_h5 (nM) - Curve 2	2.807	3.255	4.396
Intra-day relative standard deviation (%)		2.90	

A 2-ways ANOVA was performed to determine the coefficients of variation. We find that our determination of the mean R_h for each point of the curve shows an intra-day variation of 2.90%.

- Inter-day precision on the K_D of the SpikeRBD/ACE2 complex

Table S6 within-day variability of SpikeRBD/ACE2 K_D

Context of the measure	Date	T° of chip (°C)	[$SpikeRBD_{labelled}$] (nM)	[ACE2] (nM)	K_D (nM)
Proteins dissolved in PBS-T	2022/01/20	23.8	5	0.180-750	30.24
	2022/01/21	23.8	5	0.180-750	33.39
	2022/01/24	26.1	5	0.180-750	28.00
Proteins dissolved in in DMSO 1 %, v/v	2022/02/21	25.2	20	0.180-750	45.32
	2022/02/21	25.6	20	0.180-750	30.00
Mean					33.39
Standard Deviation					6.94
Relative Standard Deviation (%)					20.79

5. *Quality of curve fitting*

The data obtained during the K_D determination are sigmoidal. Applying nonlinear least-squares fitting method [3], a coefficient of determination (R^2) was calculated.

Table S7 Quality of adjustment - Determination of the R^2

Context of the measure	[$SpikeRBD_{labelled}$] (nM)	[ACE2] (nM)	R^2
K_D Determination in PBS-T - curve 1	5	0.180-750	0.8542
K_D Determination in PBS-T - curve 2	5	0.180-750	0.9227
K_D Determination in PBS-T - curve 3	5	0.180-750	0.943
K_D Determination in DMSO 1 % v/v - Curve 1	20	0.180-750	0.9423
K_D Determination in DMSO 1 % v/v - Curve 2	20	0.180-750	0.9626
Mean			0.925
Standard Deviation			0.042

6. *References*

1. Fluidic Analytics. Available online: https://www.fluidic.com/resources/hydrodynamic-radius-and-protein-weight/ [Accessed 09 april 2022].
2. Fluidic Analytics. Available online: https://www.fluidic.com/calculators-page/ [Accessed 23 october 2023].
3. FIEDLER, S., PIZIORSKA, M. A., DENNINGER, V., MORGUNOV, A. S., ILSLEY, A., MALIK, A. Y., SCHNEIDER, M. M., DEVENISH, S. R. A., MEISL, G., KOSMOLIAPTSIS, V., AGUZZI, A., FIEGLER, H. & KNOWLES, T. P. J. Antibody Affinity Governs the Inhibition of SARS-CoV-2 Spike/ACE2 Binding in Patient Serum. *ACS Infectious Diseases* **2021***,* 7**:** 2362-2369. doi:10.1021/acsinfecdis.1c00047
